# Impact of the COVID-19 pandemic on neuroendocrine tumour services in England

**DOI:** 10.1007/s12020-020-02564-2

**Published:** 2021-01-05

**Authors:** Martin O. Weickert, Tim Robbins, Ioannis Kyrou, Adam Hopper, Eilish Pearson, Thomas M. Barber, Gregory Kaltsas, Harpal S. Randeva

**Affiliations:** 1 The ARDEN NET Centre, ENETS Centre of Excellence, University Hospitals Coventry & Warwickshire NHS Trust, Clifford Bridge Road, Coventry, CV2 DX UK; 2 Warwick Medical School, University of Warwick, Coventry, CV4 7AL UK; 3 Centre of Applied Biological & Exercise Sciences, Faculty of Health & Life Sciences, Coventry University, Coventry, UK; 4 Institute of Digital Healthcare, WMG, University of Warwick, Coventry, CV4 7AL UK; 5 Aston Medical Research Institute, Aston Medical School, Aston University, Birmingham, B4 7ET UK; 6 National and Kapodistrian University of Athens, Athens, Greece

**Keywords:** COVID-19, Neuroendocrine tumours, Centres of Excellence, Service disruption

## Abstract

**Purpose:**

During the COVID-19 pandemic, there have been particular concerns regarding the related impact on specialist tumour services. Neuroendocrine tumour (NET) services are delivered in a highly specialised setting, typically delivered in a small number of centres that fulfil specific criteria as defined by the European Neuroendocrine Tumour Society (ENETS). We aimed to address the COVID-19-related impact on specialist NET tumour services in England and other countries.

**Methods:**

Electronic survey addressing patient access and delivery of care distributed to all ENETS Centres of Excellence (CoE) in England and matching number of ENETS CoE elsewhere. Semi-quantitative and qualitative analyses of survey responses were performed.

**Results:**

Survey response of ENETS CoE in England was 55% (6/11). Responses from six non-UK ENETS CoE elsewhere were received and analysed in a similar manner. Relevant disruption of various NET services was noted across all responding Centres, which included delayed patient appointments and investigations, reduced availability of treatment modalities including delayed surgical treatment and a major negative impact on research activities. The comparison between English and non-UK ENETS CoE suggested that the former had significantly greater concerns related to future research funding (*p* = 0.014), whilst having less disruption to multidisciplinary meetings (*p* = 0.01). A trend was also noted towards virtual patient appointments in ENETS CoE in England vs. elsewhere (*p* = 0.092).

**Conclusions:**

Restoration of highly specialised NET services following COVID-19 and planning for future service delivery and research funding must take account of the severe challenges encountered during the pandemic.

## Introduction

COVID-19 was first described amongst a cluster of patients suffering from pneumonia in Wuhan, China [1]. Fuelled by its viral novelty, high infectivity and global mobility [2], COVID-19 rapidly spread around the world, and within just 3 months became a global pandemic. As a rapidly evolving and dynamic entity, the true global implications of COVID-19 remain unknown, and will probably only become clear in many years to come. As per 12th of November 2020, at least 1.27 million people have died from COVID-19. The tsunami-like ramifications of COVID-19 and the associated need for ‘lockdown’ and ‘social distancing’ have had a devastating impact on the global economy that is unprecedented in the modern era [3].

COVID-19 has also had a unique and devastating impact on global healthcare infrastructures and administration, with multiple contributing factors. Although there are inevitable direct effects on service provision from increased hospital including intensive care admissions of patients with COVID-19, it is perhaps the multiple and insidious indirect effects of the COVID-19 pandemic that have most affected healthcare. During the COVID-19 era, there has been a dramatic reduction in the usage of NHS-based services [4, 5], potential explanations including patient-based risk perceptions and the impact of government-based messages regarding lockdown and self-isolation [5]. Coupled with this has been healthcare staff redeployment in many hospitals to manage the surge in hospital admissions with COVID-19, coupled with re-purposing of clinical areas for COVID-19 screening, with an associated and inevitable disruption to the provision of non-COVID-19 based, but nonetheless essential healthcare. To address these multiple and complex factors, much healthcare provision has been administered through remote media, such as phone calls and telehealth [6]. The versatility and convenience of such remote healthcare provision promotes its likely ongoing usage for at least some aspects of service in the post-COVID-19 era.

Of particular concern has been the impact of COVID-19 on tumour services, including the impact of delayed and missed clinic appointments for the overall health of the populace [7, 8]. Healthcare provision for neuroendocrine tumours (NETs) represents a specialised form of services that is typically administered by highly skilled multidisciplinary teams operating within s Centres of Excellence (CoE). Recently, guidance for the management of patients with NETs during the COVID-19 era was published [9–11]. Given the nature of NET-based clinical services, the relatively small number of NET-based clinical centres and the vulnerability of many patients with NETs, it is important to explore the impact of the COVID-19 pandemic on patients with NETs, and the associated specialist provision of healthcare.

Our aim was to perform an assessment on the impact of COVID-19 on healthcare administration and care for patients with NETs, with cross-national comparisons between European Neuroendocrine Tumour Society (ENETS) CoE based in England and those in other countries.

## Materials and methods

An electronic survey was distributed to all 11 ENETS CoE in England, via e-mail. A similar number of non-UK ENETS CoE were contacted, for comparison. The survey was designed using Google Forms and captured information in the following categories: (1) background/pre-COVID-19 state, (2) impact of COVID-19 on clinical services, (3) impact on operational service provision, (4) impact on research. Questions included in the survey comprised both semi-quantitative and qualitative response options. Qualitative analysis took the form of thematic analysis (and where appropriate, coding) of answers received. A full breakdown of the questions asked is shown in Appendix 1. The survey was analysed comparing ENETS CoE in England to ENETS CoE elsewhere, using both qualitative and semi-quantitative analysis approaches including qualitative analysis of free-text responses. Responses were received from the specialist NET healthcare professionals (Consultants/Centre leads) who consented to participate in this survey. Given that this was an international survey, ethics approval was pursued through the Institution with the shortest period of approval (Ethics Committee of the Laiko General Hospital, Athens, Greece; protocol number 10712, date of issue 6 July 2020).

### Statistical analyses

Data are presented as means ± standard error, or percentages, as appropriate. Significance was defined as *p* < 0.05. One-way analysis of variance was used to compare study subgroups. Analyses were performed using SPSS version 25 (SPSS Inc., Chicago, IL).

## Results

### Responses received

Responses received are shown in Table 1. There were 6 (55%) of responses from the 11 ENETS CoE in England. Responses from six ENETS CoE outside the UK were received and used for comparison. Two additional large specialist NET Centres in the USA and Italy were contacted, but these were excluded from the analyses based on the fact that these were not ENETS CoE, with possibly varying practices related to the management of patients with NET based on ENETS guidelines. We have not included a copy of the original data to preserve anonymity of the responses as providing such data may inadvertently allow a specific centre to be identified.

**Table 1:** Responses received from contacted European Neuroendocrine Tumour Society (ENETS) Centres of Excellence (CoE)

Country	Number of responses
England	6
Italy	1
Belgium	1
France	1
Greece	1
Israel	1
Netherlands	1

### Impact of COVID-19 in all responding ENETS CoE, all responding centres combined (*n* = 6 ENETS CoE in England and *n* = 6 ENETS CoE in other countries)

#### Impact on waiting times

The majority of ENETS CoE (58.3%, seven centres) reported an increase in waiting times for new patients with NET, of those 25% (three centres) reported an increase in waiting times of >1 week, 16.7% (two centres) reported an increase in waiting times > 2 weeks, and 16.7% (two centres) reported an increase in waiting times > 1 month. Delays in follow-up for patients with NET were reported in almost all responding centres (11 centres, 91.7%), of those 4 centres (33.3%) reported delays < 1 month, 5 centres (41.7%) reported delays > 1 month, and 2 centres (16.7%) reported delays > 2 months. Interestingly, one of the ENETS CoE in England (8.3%) reported improved follow-up waiting times; however, the total number of patients with NET in this centre was relatively small (*n* = 150) when compared to typical numbers in other ENETS CoE (*n* = 487 ± 200 patients in all centres combined; *n* = 647 ± 384 patients in the centres in England; and 327 ± 133 patients in non-UK Centres).

#### Impact on diagnostic services

All of the surveyed ENETS CoE (12 centres, 100%) reported delays on Endoscopy services, of those 1 centre (8.3%) reported delays < 1 month, 4 centres (33.3%) reported delays > 1 month, 6 centres (50%) reported delays > 2 months, and 1 centre (8.3%) reported that Endoscopy services were on halt. Only one centre (8.3%) reported that there was no impact on morphological imaging, whereas two centres (16.7%) reported delays < 1 month, four centres reported delays > 1 month, and five centres reported delays > 2 months. Delays on blood test monitoring for patients with NET during COVID-19 were reported by 83.3% of the centres, of those four centres (33.3%) reported small delays < 1 month, three centres reported large delays > 1 month, and three centres reported very large delays > 2 months.

#### Impact on treatment

Impact on the provision of specialist medication such as self-administered somatostatin analogues, mTOR inhibitors or telotristat etiprate was reported by four centres (33.3%), but in all cases the delay was reported as small (<2 weeks). However, as many as six ENETS CoE (50% of the surveyed centres) reported difficulties in the availability of specific medications such as chemotherapy and interventional radiological treatment (i.e., transarterial embolization or radiofrequency ablation). A negative impact on surgical treatment was reported by 11 centres (91.7%), of those 8 centres (66.7%) reported large delays > 1 month and 3 centres (25%) reported very large delays > 2 months. Impact on the provision of peptide receptor radionuclide therapy (PRRT) was reported by ten of the centres (83.3%), of those four centres (33.3%) reported small delays < 1 month, three centres (25%) reported large delays > 1 month, two centres reported very large delays > 2 months, and one centre (8.3%) reported that PRRT was on halt. Only two of the centres (16.7%) reported that there was no disruption to the provision of PRRT.

#### Change of face-to-face to virtual appointments

All 12 centres (100%) reported a move from face-to-face to virtual appointments, of those 2 centres (16.7%) reported that the minority of appointments were changed to virtual, 9 centres (75%) reported that the majority of appointments were changed, and 1 of the centres (8.3%) reported that all appointments were changed to virtual appointments. The qualitative responses regarding clinician perceptions to remote consultations were diverse (Table 2).

**Table 2:** Thematic analysis of clinician perceptions regarding remote/virtual consultations

Theme identified regarding use of virtual/remote consultations	Number of respondents
Generally advantageous	5
Inability to clinically examine patients	2
Disadvantageous to patients if already visiting for bloods, etc.	1
Can’t understand feelings/needs of patient	1
Trial recruitment is more difficult	1
Fewer patients missing appointments (DNA’s)	1
Advantageous as reduced travel need for patients	1

#### Redeployment of staff

Nursing staff was redeployed in ten centres (83.3%), of those five centres (41.7%) reported that the minority of nursing staff was redeployed, four centres (33.3%) reported major nursing staff redeployment and one centre (8.3%) reported that all nursing staff was redeployed.

Redeployment of medical staff (doctors) was reported by all 12 centres (100%), of those 8 centres (66.7%) reported minor redeployment, 3 centres (25%) reported that the majority of medical staff was redeployed and 1 centre (8.3%) reported that all medical staff was redeployed.

#### Impact on NET tumour board meetings

A negative impact on the frequency of NET tumour board meetings was reported by four of the centres (33.3%), whereas eight of the centres (66.7%) reported no change. A move to virtual meetings was reported by five of the surveyed centres (41.7%).

#### Impact on research and research funding

Only one of the centres (8.3%) reported that there was no impact of the COVID-19 pandemic on research activity. Some reduction in research activity was reported by four centres (33.3%), while six of the centres (50%) reported very significant reduction in research activity and one centre (8.3%) reported that all research activity was stopped.

The perceived impact of the COVID-19 pandemic on the availability of future research funding for the NET service in the respective centres was reported as no impact by four of the centres (33.3%), an expected small reduction in research funding availability by two of the centres (16.7%), a large reduction by four of the centres (33.3%) and a very large reduction by two of the centres (16.7%).

Qualitative themes raised in relation to long-term changes to NET services included: fewer face-to-face appointments, longer waiting times, long-term impacts on treatment and reductions in the number of staff.

### Comparison of ENETS CoE in England vs. ENETS CoE in other countries

When comparing the effects of the COVID-19 pandemic on the responding six ENETS CoE in England vs. six ENETS CoE in other countries, statistically significant differences were observed related to the frequency of tumour board meetings (*p* = 0.01), with none of the ENETS CoE in England reporting a decrease in the frequency of tumour board meetings, whereas four of the ENETS CoE elsewhere (66.7%) reported a decrease in the frequency of tumour board meetings. Delay in surgical treatment or treatment with PRRT was observed in most of the participating ENETS CoE, with no significant differences between ENETS CoE in England and elsewhere (*p* = 0.33 (surgical treatment) and *p* = 0.66 (PRRT), respectively).

Analysis of the perception of the impact of the pandemic on the availability of future research funding showed that all the surveyed six centres in England (100%) expected a reduction in funding, of those two centres (33.3%) a small reduction, two centres (33.3%) a large reduction and two centres (33.3%) a very large reduction in available NET-related research funding. In comparison, only two of the ENETS CoE elsewhere (33.3%) expected a large reduction in research funding availability, whereas four of the non-England ENETS CoE expected no impact of the COVID-19 pandemic on NET research funding availability (*p* = 0.014). No other significant differences were observed when comparing ENETS CoE in England vs. ENETS CoE elsewhere. Related to a move from face-to-face to virtual patient appointments, this practice tended to be more frequent in ENETS CoE in England as compared with ENETS CoE in other countries (*p* = 0.092).

## Discussion

Our study is capturing ‘real life’ perceptions about the impact of the current COVID-19 pandemic on specialist NET services in ENETS CoE. In contrast to other recently published studies that had also included ‘low-activity’ (<100 neuroendocrine neoplasms (NEN) patients in follow-up) [12, 13] and ‘mid-activity’ centres (100–300 NEN patients in follow-up) [13], in our study we have exclusively focussed on the effects of the COVID-19 pandemic in typically ‘high activity’, University or University Teaching Hospital-based ENETS CoE. Our present findings demonstrate a significant disruption to NET services across all surveyed centres. Of note, this major disruption in specialist NET services included relevantly increased waiting times for both new and follow-up appointments, with an especially substantial delay of follow-up appointments in most of the surveyed centres. Similar findings were reported by a recent study in Italian NEN centres [13] including also ‘low-’ and ‘mid-activity’ centres, whereas in a recent study in Germany, Austria and Switzerland disruption in outpatient appointments was more severe in the University setting as compared with non-university hospitals and private practice settings [12]. We also observed a relevant disruption to diagnostic services. Similarly, specialised treatment of patients with NETs was delayed, which included a relevant delay in surgical treatment; and again, in agreement with the observations made in other countries such as Italy [13]. In our survey, this included all types of NET, independent of the primary location and histological grading. Although many NETs are relatively slow growing, a delay in curative surgical treatment may result in disease spreading and clearly has the potential to increase anxiety levels in these patients, thereby further negatively impacting their quality of life [14].

A COVID-19 pandemic-related delay in treatment with PRRT based on concerns, i.e., due to the frequently observed PRRT-associated lymphopenia may be somewhat overcautious, given that PRRT mainly appears to cause B-cell repletion (in 18–52% of patients treated with ^177^Lutetium-PRRT and some 75% of patients treated with ^90^Yttrium-PRRT) [15, 16], but appears to have less severe effects on T cells and only minor effects on natural killer cells, explaining the absence of opportunistic infections following treatment with PRRT [15]. However, both for planned surgical interventions and treatment with PRRT, hospital beds need to be ‘ring-fenced’, which can be problematic in times of a pandemic with overflowing Acute Medicine and A&E departments and the need to isolate numerous patients in one bed rooms.

Moreover, we observed a change in practice in the way of both new patient and follow-up appointments are provided, with all surveyed centres now providing more virtual appointments and most centres reporting that the majority of appointments had been converted to virtual appointments. This change in practice was more frequently reported in the ENETS CoE in England vs. other countries, possibly related to the availability and fast set-up of the required equipment. Feedback of clinicians to this change was mixed, with the main disadvantages mentioned being unable to physically examine the patients. However, perceived positive aspects included improved convenience for (stable) patients who do not live locally, and a reduced number of non-attenders.

Redeployment of both nursing and medical staff was reported in most of the surveyed centres, but the impact on the management of patients with NET was generally considered to be relatively minor. A reduction in the frequency of multidisciplinary meetings was reported in two thirds of the ENETS CoE outside England, but no such effect was reported in the ENETS CoE in England. However, across the surveyed centres nearly half of the centres now run their tumour board meetings virtually. The feedback of clinicians to this change was generally positive, although there can be barriers related to connection issues and the quality of the available equipment.

All but one of the non-UK centres reported that there was a negative impact of the COVID-19 pandemic on research activity. There was no difference in the disruption of research activity when comparing ENETS CoE in England vs. ENETS CoE elsewhere. However, the perceived impact of the COVID-19 pandemic on the availability of future research funding for the NET services in the respective centres was bleaker in the English ENETS CoE and significantly different between ENETS CoE in England and elsewhere. Our results suggest that ENETS CoE in England may face a longer road to restoration of NET services due to reduced research activity and particularly pessimistic predictions regarding future research funding. The impact on research funding is further important as it may coincide with greater funding difficulties related to the UK’s departure from the European Union (Brexit) [17–19], suggesting that very significant efforts and mitigations would be required UK NET research centres now, to ensure that they can fully contribute to research in the future.

A limitation of our survey is that the data presented here, albeit novel and detailed, is from a small sample size of specialised ENETS CoE. Also, reporting bias cannot be excluded, i.e., some of the NET Centres worst affected by the COVID-19 pandemic may simply not have prioritised responding to our survey invitation. Finally, the fact that only one centre response for each of the other countries was received (also having in mind that in some of the contacted countries, only one ENETS CoE has been certified to date) is limiting how much one can interpret the data for individual countries other than England. The results should be interpreted in that context. However, despite these limitations, we believe there are important messages for both restoration of services and planning of future NET care and research approaches. Overall, it is important that such specialist areas are not being neglected in comparison to broader services. There is important further work that is needed to monitor and track the recovery of all services including NET services and research following the COVID-19 pandemic. Furthermore, it is important to assess the impact of COVID-19 and the related healthcare disruptions to services on patients themselves, both by capturing their perceptions of care during the pandemic and by monitoring for any worsening outcomes that may have occurred. One of the possible positives that appears to come from the pandemic is an exploration of the use of virtual and remote services.

To conclude, we report on the first study to assess the impact of COVID-19 on ENETS CoEs in England, and compare with data from a selection of ENETS CoEs from other western European countries. This survey has identified various deficiencies in the provision of NET services even in highly specialised set-up, as a result of the COVID-19 pandemic. The same may apply for tumour services in general, although it is possible that highly specialised services with relatively low numbers of patients could be particularly affected. Future assessment of the impact of COVID-19 on NET healthcare from a patient perspective is important. Based on our data, it is important to develop novel and unified approaches to future healthcare provision for patients with NETs. This may include critical review of possible widespread adoption of remote appointments and proper planning for future pandemic scenarios that minimises disruption to the provision of healthcare to this important and vulnerable group of patients with unique and highly specialised medical needs.

## Supplementary information

MOW_NET Appendix 1_COVID_survey
