# Supplementary material for: Impact of the COVID-19 pandemic on neuroendocrine tumour services in England
Source: Endocrine. 2021 Jan 5;71(1):14–9. doi: 10.1007/s12020-020-02564-2 (PMC7782563; doi:10.1007/s12020-020-02564-2)
Supplement: Supplementary file 1 — MOW_NET Appendix 1_COVID_survey [file 12020_2020_2564_MOESM1_ESM.pdf]

Impact of COVID-19 on International Neuroendocrine (NET) services.

This survey looks to better understand the impact of COVID-19 on ENETS Centres of excellence internationally. This will support both restoration work and the ability to better mitigate future crises. We anticipate the survey will take up to 10 minutes of your time.

The survey is optional, data will be collected and stored for the above purposes. Data is collected using GoogeForms. If you do not wish to participate in the survey, please close your browser now.

Background Questions

1. Are you an ENETS Centre of Excellence?

   Mark only one oval.

   - Yes
   - No

2. What country is your NETS Centre based in?

   ____

3. How many NET patients do you care for per full time medical doctor?

   ____

4. Please describe your typical (pre-COVID) follow up plans for high risk and low risk patents

   ____

Impact of COVID-19 on Clinical Services

5. What has been the impact on waiting times for new patients?

   Mark only one oval.

   - Very large increase in waiting times (Delay over 1 month)
   - Large increase in waiting times (Delay over 2 weeks)
   - Some increase in waiting times (Delay over 1 week)
   - No change
   - Faster review of new patients

6. What has been the impact on follow up times for patients

   Mark only one oval.

   - No follow ups occured during COVID-19
   - Very large delays in follow up (delay over 2 months)
   - Large dealys in follow up (delays over 1 month)
   - Small dealys in follow up (delays less than one month)
   - No change
   - Increased follow up of patients

7. What has been the impact on endoscopy services for patients?

Mark only one oval.

- No endoscopy available during COVID-19
- Very large delays in endoscopy (delays over 2 months)
- Large delays in endoscopy (delays over 1 month)
- Small delays in endoscopy (delays less than one month)
- No change
- Greater availability of endoscopy

8. What has been the impact on morphological imaging for patients?

Mark only one oval.

- No morphological imaging available during COVID-19
- Very large delays in morphological imaging during COVID-19 (delays over 2 months)
- Large delays in morphological imaging during COVID-19 (delays over 1 monthy)
- Small delays in morphological imaging during COVID-19 (delays less than 1 month)
- No change
- Greater availability of morphological imaging

9. What has been the impact on blood test monitoring for patients during COVID-19

Mark only one oval.

- No blood test monitoring available during COVID-19
- Very large delays in blood test monitoring (delays over 2 months)
- Large delays in blood teset monitoring (delays over 1 month)
- Small delays in blood test monitoring (delays less than one month)
- No change
- Greater availability of blood test monitoring

10. What has been the impact on medication provision for patients during COVID-19 (ie SSA, molecular targeted & telotristat that can be administered by the patients themselves)

Mark only one oval.

- Very large delays in providing medication (delays over 1 months)
- Large delays in providing medication (delays over 2 weeks)
- Small delays in providing medication (delays less than 2 weeks)
- No change

11. Have you had difficulties in the availability of specific medications such as chemotherapy and interventional 'TAE, SIRT, RF.

Mark only one oval.

- Yes
- No
- Other: ______________________

12. What has been the impact on surgical intervention during COVID-19 for NET patients

Mark only one oval.

- Very large delays in surgery (delays over 2 months)
- Large delays in surgery (delays over 1 month)
- Small delays in surgery (delays less than 2 weeks)
- No change
- No surgery available
- Greater availability of surgery

13. What has been the impact on Peptide receptor radionuclide therapy during COVID-19 for NET patients

Mark only one oval.

- Very large delays in PRRT (delays over 2 months)
- Large delays in PRRT (delays over 1 month)
- Small delays in PRRT (delays less than 2 weeks)
- No change
- No PRRT available
- Greater availability of PRRT

14. What has been the impact on the management of acute problems (ie management of side effects) during COVID-19?

Impact on operational service provision

15. To what extent have your services been moved from face to face consultations to virtual consultations (via video or telephone).

Mark only one oval.

- All appointmens are (or were) moved to remote consultations
- The majority of appointments are (or were) moved to remote consultations
- The minority of appointments are (or were) moved to remote consultations
- No change in face-face offering of services

16. How appropriate do you feel virtual/remote consultations are for your NET service and the patients you treat? Please describe your opinion of the advantages and disadvantages?

17. To what extent have your nursing staff teams been redeployed to support COVID-19 care?

Mark only one oval.

- All nursing staff redeployed
- The majority of nursing staff redeployed
- The minority of nursing staff redeployed
- No nursing redeployment

18. To what extent have your medical staff (doctors) been redeployed to support COVID-19 care.

Mark only one oval.

- All medical staff redeployed
- Majority of medical staff redeployed
- Minority of medical staff redelopyed
- No redeployment

19. Do you have any comments about redeployment of medical and nursing staff, the appropriateness of their skills for redeployment and/or the impact on patients?

20. What have you done (or been able to do) to mitigate the impact of COVID-19 on your NET service?

21. How long do you think it will take for clinical provision for NET patients in your area to return to normal or a "new normal" state?

22. Do you think there will be any long lasting impacts from COVID-19 on your service (please include whether you think these will be positive or negative)?

23. What has been the impact of COVID-19 on the timing of tumour board / MDT meetings?

Mark only one oval.

- All tumour board / MDT meetings cancelled
- Reduction in the number of tumour board / MDT meetings
- No change in timings of tumour board / MDT meetings

24. Please describe any other changes in tumour board / MDT meetings as a consequence of COVID-19, and their impact on patients.

Impact on Research

25. What has been the impact of COVID-19 on NETS related Research?

Mark only one oval.

- All research stopped
- Very significant impact on research
- Some reduction in research activity
- No impact on research activity

26. When do you think NETS research activity will be able to return to normal following COVID-19?

27. What do you think the impact of COVID-19 will be on the availability of future research funding for the NET service at your centre?

Mark only one oval.

- Very large reduction in research funding availabilty
- Large reduction in research funding availability
- Small reduction in research funding availability
- No impact in research funding availability
- Small increase in research funding availability
- Large increase in research funding availability
- Very large increase in research funding availability

Follow up short interview

28. We would like to follow up this survey with a short (10-15 minute telephone interview). If you would be happy to participate please could you include your telephone number (including international dialling code) and email address below. This will only be used for the purposes of conducting the interview. If you would prefer not to include your details associated with your survey responses please email Prof Kaltsas & Prof Weickert using the address: martin.weickert@uhcw.nhs.uk and/or gkaltsas@endo.gr

This content is neither created nor endorsed by Google.

Google Forms
